# A Culturally Sensitive Mobile App (DiaFriend) to Improve Self-Care in Patients With Type 2 Diabetes: Development Study

**DOI:** 10.2196/63393

**Published:** 2024-10-21

**Authors:** Peeranuch LeSeure, Elizabeth Chin, Shelley Zhang

**Affiliations:** 1 College of Nursing and Health Sciences University of Massachusetts North Dartmouth, MA United States; 2 Department of Mathematical, Computer, and Data Science Gordon College Wenham, MA United States

**Keywords:** cultural sensitivity, design thinking, mobile app, self-care, type 2 diabetes, mobile phone, app, design, diabetes, development, prototype, effectiveness, user engagement, blood glucose, glucose, weight, carbohydrate, exercise, Portuguese Americans, ethnic group

## Abstract

**Background:**

Mobile apps designed with cultural sensitivity have demonstrated higher user acceptability and greater effectiveness in enhancing self-care skills. However, a significant gap exists in developing such apps for specific populations, such as Portuguese Americans living in southern Massachusetts, home to the second-largest Portuguese community in the United States. This group possesses unique cultural traditions, particularly in dietary practices, including a tendency toward high carbohydrate intake. Tailoring diabetes self-care apps to address these specific cultural requirements could substantially improve diabetes management within this population.

**Objective:**

The aim of this app development project was to develop a prototype diabetes management app for Portuguese Americans with type 2 diabetes mellitus using the design thinking methodology, incorporating user-centered design principles and cultural sensitivity. This paper describes the phase-2 results, focusing on app design and development.

**Methods:**

Phase 2 of this app development project adhered to the design thinking methodology delineated by the Hasso Plattner Institute of Design at Stanford University, focusing on 2 critical steps: ideation and prototyping. This phase started in March 2022 and continued until April 2024. The project was driven by a multidisciplinary team consisting of 2 nurse educators; an app development specialist; and 2 graduate research assistants from the university’s Computer and Information Sciences Department, both well-versed in mobile app development. Data collected during phase 1, which will be published separately, informed the app design and development process.

**Results:**

The prototype of the DiaFriend app (version 1) was designed and developed. The app comprises five features: (1) blood glucose monitoring, (2) weight tracking, (3) carbohydrate tracking, (4) exercise log, and (5) medication reminder. The carbohydrate tracking feature was explicitly tailored to correspond to Portuguese food culture. This paper presents the front-end interface flowchart, demonstrating how the user navigates through each screen. It also discusses the challenges faced during the backend development, such as data not being able to be stored and retrieved.

**Conclusions:**

The DiaFriend app (version 1) distinguishes itself from conventional diabetes self-care apps through its emphasis on cultural sensitivity. The development of this app underscores the importance of cultural considerations in health informatics. It establishes a foundation for future research in developing and evaluating culturally sensitive mobile health apps. The adaptation of such technologies has the potential to enhance self-care practices among Portuguese Americans with type 2 diabetes mellitus, with improved glycated hemoglobin levels as a potential outcome. The last step of the design thinking methodology, testing the app, will be conducted in phase 3 and the results will be published elsewhere.

## Introduction

The number of adults aged 18 years or older with diabetes in the United States reached 38.4 million, or 11.6% of all US adults in 2021 [1]. Between 2002 and 2018, the overall incidence of diabetes significantly increased, with approximately 16.8 million adults aged 18 years or older with diabetes visiting the emergency department, including 267,000 for hyperglycemic crisis (11.4 per 1000 adults) and 202,000 for hypoglycemia (8.6 per 1000 adults) [2].

Approximately 1.5 million people of Portuguese descent live in the United States, with over 265,000 residing in Massachusetts, which has the second-highest concentration of Portuguese Americans [3]. Despite being one of the largest ethnic groups in Massachusetts and several other states, the health status, issues, and disparity among the Portuguese American population are not well documented [4]. However, statistical data indicate a significantly higher prevalence of diabetes among numerous racial and ethnic minority populations in the United States relative to non-Hispanic White Americans [3]. In Massachusetts, 14.7% of people aged from 45 to 64 have diabetes [5]. In the South Coast region of Massachusetts, particularly in New Bedford and Fall River, cities with substantial Portuguese American populations, the prevalence of diabetes among adults aged 18 and older exceeds the national average [6]. Moreover, diabetes ranks among the sixth leading causes of death in this area, with mortality rates showing an annual increase [6].

Portuguese Americans face unique challenges in managing type 2 diabetes mellitus (T2DM) and preventing its complications due to their cultural traditions, particularly dietary practices. The carbohydrate-rich, fatty, and sugary nature of many traditional Portuguese dishes, desserts, and pastries may increase the risk of developing diabetes. These dietary traditions pose significant obstacles for Portuguese Americans in adopting and maintaining the self-care behaviors necessary for effective diabetes management.

Self-care is considered essential in chronic illness management. Riegel et al [7] defined self-care as a process of maintaining health through health promotion practice and managing illness. T2DM is a complex chronic condition that requires ongoing self-care behavior modification, with patients assuming an active role in making daily decisions that impact their health and well-being. Effective diabetes management requires substantial lifestyle modifications with physical and psychological adaptation to maintain healthy behaviors. Mobile apps have provided support for diabetes self-care in patients with T2DM, leading to enhanced glycemic control [8-11]. Mobile apps offer significant potential to enhance diabetes self-management by providing patients with multifaceted support. These digital tools facilitate convenient blood glucose monitoring, carbohydrate tracking, physical activity promotion, access to educational resources, and personalized guidance. However, many existing apps lack user-friendly interfaces and personalization of features, fail to accommodate diverse cultural perspectives, and do not offer experiences tailored to individual needs and preferences [12-14].

In total, 2 studies have addressed the importance of culturally sensitive apps for diabetes management to improve blood glucose in Asian people with diabetes [14,15]. They identified the gap in culturally tailored content and features that address specific dietary habits, lifestyle practices, and health beliefs of diverse ethnic populations. In the first study, the Welltang app, a smartphone-based diabetes management app tailored to Chinese culture, demonstrated significant positive outcomes among its users, including reductions in glycated hemoglobin (HbA_1c_) levels and blood glucose concentrations, alongside increased satisfaction with the app’s functionality [15].

The second study used qualitative methodologies to design a prototype app aimed at enhancing diabetes self-care behaviors and self-management outcomes [14]. The researchers identified a significant gap in culturally sensitive diabetes apps tailored to Asian populations. Consequently, they conducted a qualitative study involving patients and health care experts in app development. However, the results of this study have not yet been published, and this study, highlighting the need for culturally tailored weight control apps for Hispanic and Brazilian Americans, provides compelling evidence for the importance of culturally sensitive apps in improving self-care. This research is particularly relevant to diabetes management, as it addresses health-promoting behaviors such as healthy eating and physical activity, which are crucial components of diabetes self-care. The study’s findings underscore the potential impact of culturally adapted digital tools in enhancing self-management practices across various health domains.

In addition to a lack of cultural sensitivity, other obstacles to using diabetes apps include time-consuming multistep tasks, repetitive data-entry processes, and complicated system navigation, all of which require technological skills. These factors could pose significant barriers for people with T2DM to adopt and integrate apps into their daily lives. To compound these challenges, individuals who are not technologically savvy or who face socioeconomic disadvantages, as well as those from diverse racial and ethnic backgrounds, may find these apps to be obstacles rather than aids in improving self-care behaviors and supporting self-management of T2DM [8,16-19].

While mobile apps have the potential to provide significant benefits for diabetes self-management, current research reveals critical limitations. Many existing apps lack cultural sensitivity specific to users’ ethnic backgrounds, and their features often require advanced technological skills. These factors may significantly diminish the effectiveness and adoption rates of apps. Despite recognizing these issues, a significant gap remains in the literature regarding developing culturally tailored diabetes management apps for diverse populations. Portuguese Americans with T2DM have distinct preferences that influence their behavior and expectations regarding technology; therefore, they require a user-centered app customized and sensitive to their culture.

The design thinking methodology, a user-centered approach that prioritizes cultural sensitivity, has recently gained popularity in mobile app development to enhance user engagement and improve self-management outcomes [9,20,21]. This methodology involves users developing an app that better meets their needs and expectations. To date, there are no culturally tailored apps for diabetes self-care among Portuguese Americans with T2DM. This app development project aimed to develop a prototype app for Portuguese Americans with T2DM using the design thinking approach.

## Methods

### App Development Design

The multidisciplinary research team consisted of 2 nurse educators, an app development expert, and 2 graduate research assistants with mobile app development knowledge from the university’s Computer and Information Sciences Department. The design thinking methodology developed by the Hasso Plattner Institute of Design at Stanford University (Stanford Design School) [20,21] was used to develop the DiaFriend app (Version 1) for Portuguese Americans with T2DM.

This paper describes phase 2 of the 3-phase app development, focusing on the design and development of the DiaFriend app prototype. This phase started in March 2022 and continued until April 2024. In the context of design thinking methodology, this phase encompasses 2 critical steps: ideate and prototype [22-24]. The app design and development process in this phase was informed by findings from phase 1, wherein 22 participants were interviewed to ascertain desired app features and functionalities. These insights from phase 1, which will be published separately, provided crucial direction for the prototype development in phase 2. The subsequent section delineates the app development process in detail.

### Ethical Considerations

Ethical approval for this app development project was obtained from the University of Massachusetts Dartmouth institutional review board (22.030).

## Results

The DiaFriend prototype app was designed and developed using the 2 steps of phase 2 of the design thinking methodology (ideate and prototype). During the ideating step, the multidisciplinary research team met multiple times to brainstorm and discuss ideas for the DiaFriend app framework, programming language, features, and app functions. Due to the funding of this project coinciding with the COVID-19 pandemic, the research team’s institution prohibited in-person interactions with app development participants. As a result, the information gathered during the ideation phase about the app design originated exclusively from the research team members.

Flowcharts were used to illustrate the step-by-step process of navigating the app, which is the flow of the screens the user will interact with to perform specific tasks while using the app. Throughout the iterative design process, the flowcharts underwent multiple revisions. During this process, the research team acknowledged that no idea should be dismissed as inherently wrong and that not every idea represents the optimal solution. This approach fostered a creative and open-minded environment, encouraging the exploration of diverse possibilities. Upon the culmination of this iterative design phase, the team used a consensus-driven approach to determine the optimal design framework explicitly tailored for Portuguese Americans with diabetes, our target user population.

Based on the participants’ recommendations collected during phase 1 of the app development published elsewhere, the researchers prioritized and selected 5 essential features to be incorporated into the initial version of the DiaFriend app. These features include blood glucose monitoring, weight tracking, carbohydrate tracking, exercise logging, and medication reminders. For weight tracking, the basal metabolic index was used. While more than 5 features were suggested, the research team first focused on implementing these 5 essential functionalities, intending to add more features in future iterations based on user feedback in the project’s next phase (phase 3).

**Figure 1:**
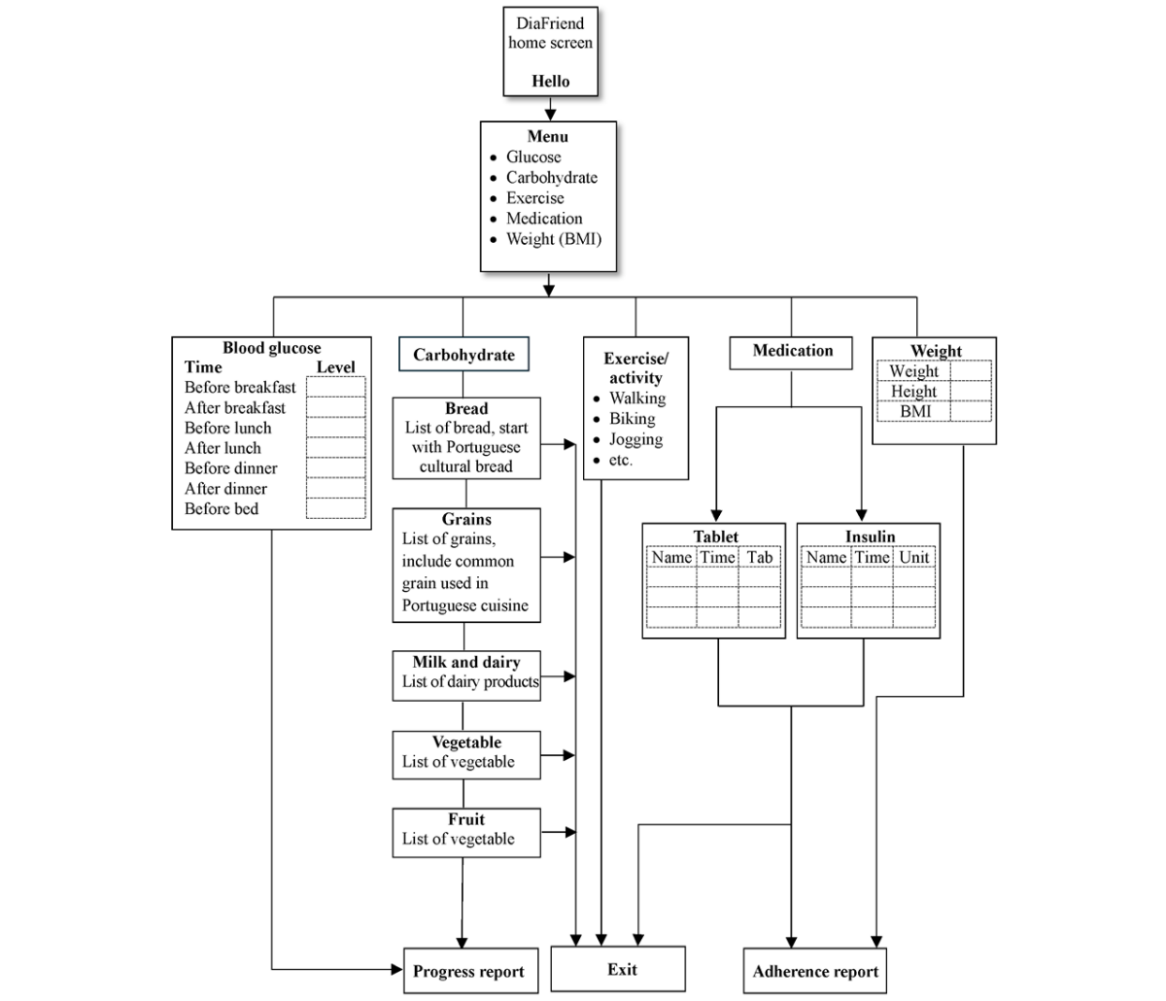
DiaFriend app flowchart.

Because the DiaFriend app does not collect and store sensitive user information, a log-in process involving a username and password was not required. This design will reduce the burden on users by eliminating the need to create and memorize a password, thereby providing a more seamless and accessible user experience. Figure 1 illustrates the tree model of the final flowchart. The flowchart begins with the entry point to the app (home page screen) and ends with the exit point, where the user completes tasks of each function.

**Figure 2:**
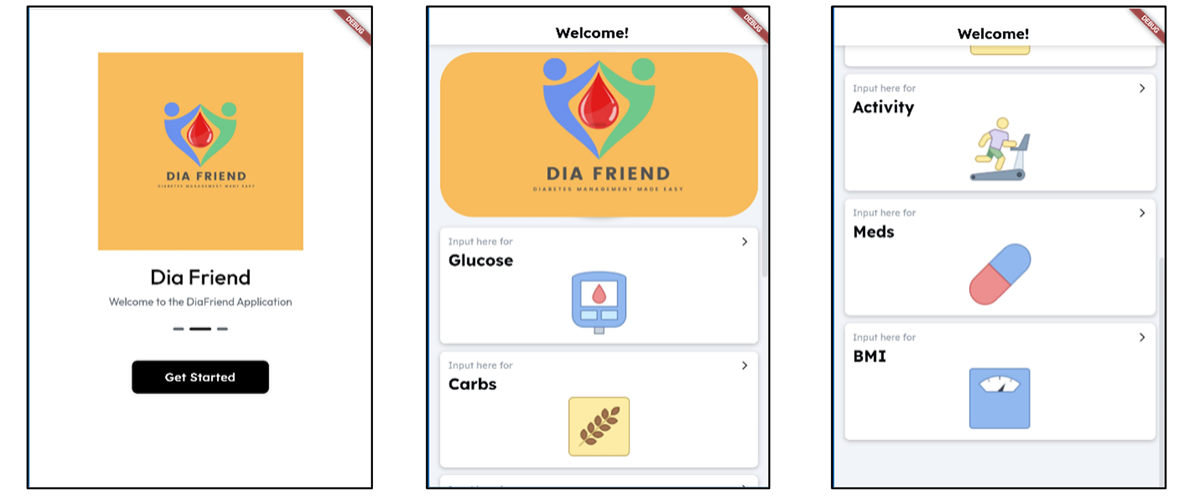
Home screen and feature screen.

In the prototype step, the prototype app development stage, the 5 features (blood glucose monitoring, weight tracking, carbohydrate tracking, exercise logging, and medication reminders) were built into the app. Flutter Software (version 2.2; Google), a single codebase, was used to build the DiaFriend prototype app; therefore, the app can run on both Android and iOS smartphones. In adherence to intellectual property rights, the research team designed and created the app logo and graphic symbols for each feature (Figure 2).

**Figure 3:**
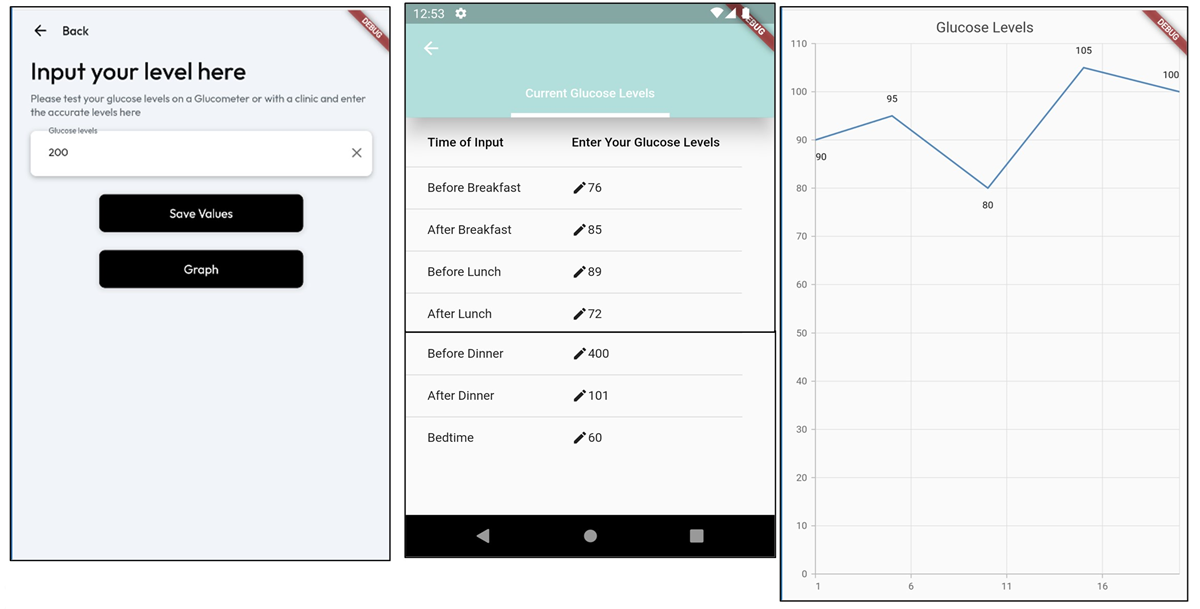
Glucose monitoring feature screen.

The glucose monitoring feature, which included a customizable list of testing times, enabled users to select the times recommended by their physicians and track their blood sugar levels in real time. As suggested in phase 1 of the app development, the input can be presented as a comprehensive list of all the results in numerical format or as a visual graph (Figure 3).

**Figure 4:**
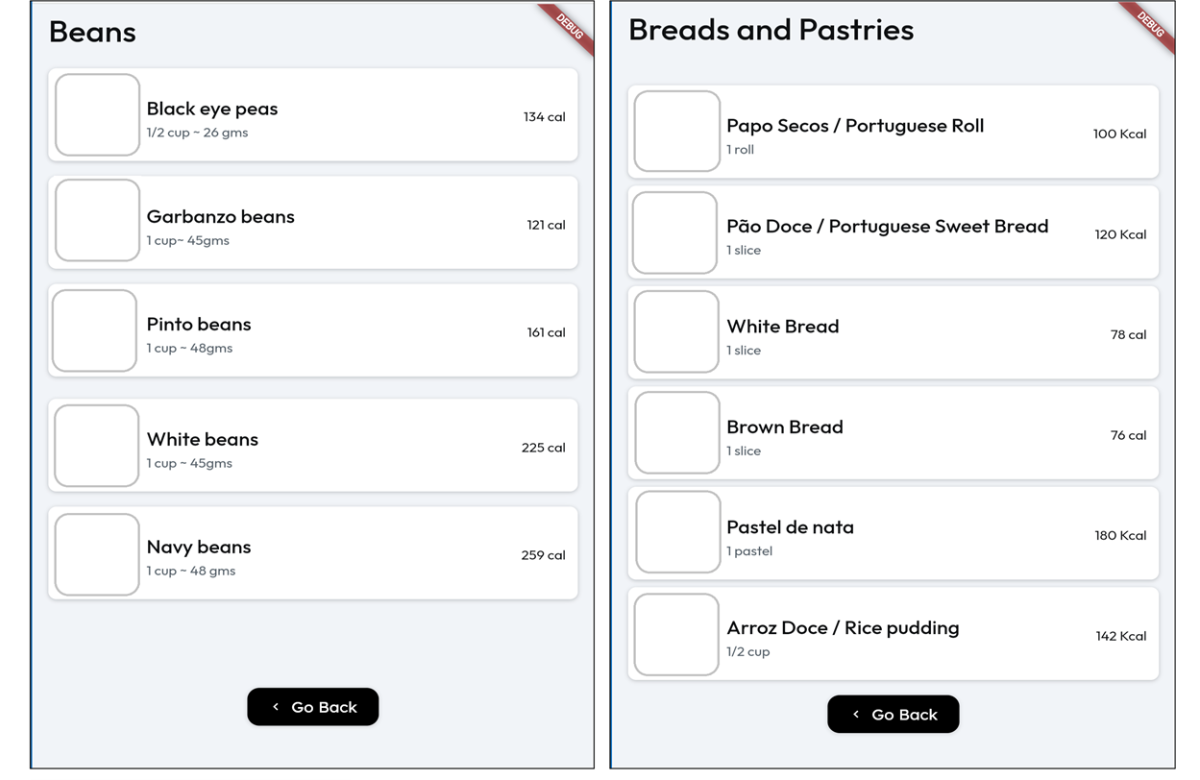
Food choice.

The carbohydrate tracking feature included food databases tailored to Portuguese food culture, incorporating Portuguese American cultural dietary choices such as various types of Portuguese bread, beans, sausages, pastries, and desserts. The database provided nutritional information (eg, calories, carbohydrates, etc) for each food item, enabling users to make informed decisions about their dietary intake. To enhance the user experience and make food selection more accessible, the app displayed pictures alongside the food names, offering visual cues that facilitate quicker recognition and choice compared with a text-only list (Figure 4). Moreover, fresh and dried figs, which are not commonly used in American cuisine but are staples in Portuguese dishes, were added to the food database to make it easier for users to track their calorie intake. The DiaFriend app incorporates various images to enhance user experience and engagement. However, due to copyright restrictions, the images used in the app cannot be reproduced or displayed in this paper.

For the exercise feature, walking was prominently featured on the exercise screen because most participants interviewed in phase 1 indicated that walking was their primary form of exercise. The research team intended to create a visually appealing and easily recognizable image design that displayed estimated calorie expenditure per mile, enabling users to make informed choices.

At this stage, the medication tracking feature permitted users to input their medication name, dosage, and time and establish reminder times. To enhance usability, the researchers plan to incorporate a prepopulated list of diabetes medications, allowing patients to select their medications without the need for manual input.

The current stage of the diabetes prototype app’s development includes a functional frontend interface that incorporates the 5 selected features. The front-end user interface was established during the initial development of the DiaFriend app; however, improvement was still necessary to ensure consistency in feature nomenclature and component design. The backend infrastructure and application programming interface still need to be developed to ensure full functionality and data management. Due to the specialized nature of backend development, the research team acknowledges the need for collaboration with experts in this field to complete the prototype. Future steps will involve partnering with experienced backend developers to design and implement the necessary backend components, such as databases, server-side logic, and communication interfaces between the front end and backend.

## Discussion

### Principal Findings

Mobile apps can effectively support self-care practices among individuals with T2DM. However, many apps lack cultural relevance, which may negatively impact user engagement, particularly within specific ethnic populations. Portuguese Americans, for instance, seem to be an underrepresented group in this context. Using 2 steps of the design thinking methodology (ideate and prototype), the research team developed the DiaFriend app, a culturally sensitive app designed to facilitate diabetes self-management among Portuguese Americans with T2DM. The DiaFriend app incorporates 5 core features: blood glucose monitoring, weight tracking, carbohydrate tracking, exercise logging, and medication reminders. As a diabetes self-care app, these features are essential for supporting effective diabetes self-management [14,19,25,26]. One of the key strengths of the DiaFriend app is its ability to integrate blood sugar management, carbohydrate tracking, and exercise monitoring into a single, user-friendly platform. Unlike existing apps that built these aspects of diabetes management separately, often requiring patients to use multiple apps to track their progress, DiaFriend consolidates all these functions into one single, user-friendly platform. Therefore, streamlining the self-care monitoring behaviors necessary for diabetes self-management into a single app should enhance user engagement and adherence by providing a solution for diabetes management [26].

Moreover, the carbohydrate tracking feature of the DiaFriend app, which incorporates Portuguese American cultural dietary choices and food images alongside the food names, is another strength of the app. T2DM is a chronic disease that requires lifelong self-care behavior modification, including dietary changes and regular exercise, often influenced by an individual’s cultural background and ethnicity. Previous studies have indicated the need for culturally sensitive and personalized apps to support self-care for specific cultural and ethnic populations [9]. Culturally tailored apps are likely to increase users’ motivation to engage in behaviors, such as caloric restriction, healthy eating, and physical activity; be perceived as personally relevant; and lead to a greater likelihood of behavior change [27]. Moreover, a dietary tracking feature should incorporate culturally relevant food databases in features with visual aids to facilitate effective monitoring of calorie intake [13]. DiaFriend app provides a carbohydrate tracking feature tailored to Portuguese food culture to ensure cultural sensitivity. Portuguese American cuisine has its specialty dishes, such as a soup made by soaking country bread in a broth; some stew containing tripe, beans, linguiça sausages, cured ham, chicken, and vegetables; and typical desserts and confections, such as flan (a baked custard topped with a caramelized sugar sauce) and pastel de nata (Portuguese custard tarts), which may be served as dessert or used as icing on a cake [28]. Therefore, the food database in the DiaFriend app includes Portuguese American cultural dietary choices from which users can select the food they consume. Furthermore, insights from phase-1 interviews revealed that walking was the most common form of physical activity among participants. Therefore, walking has been prioritized and listed first in the exercise feature, allowing users to access it immediately without scrolling down the screen.

In addition, the DiaFriend app’s user-centered design, which considers the preferences and habits of its target population, Portuguese Americans with T2DM, further distinguishes it from other apps. Previous studies indicate that a more user-friendly app is desirable; users expect less effort in learning, tracking data, and understanding the app [17]. Ease to use is one of the characteristics of diabetes self-care apps that affect patient intention to use and engage with the app, especially for participants with limited exposure to technology [12,14,16,17]. Previous studies have reported that many diabetes apps are confusing, complex, and stressful to set up and use, often resulting in a physically burdensome experience for users [13,26]. In contrast, apps tailored to individual technological skill levels are perceived as easy to use and associated with engagement and successful usability [12]. When designing the DiaFriend app, the research team adhered to the characteristics of an easy-to-use app described by Portuguese Americans with T2DM. The user interface screens were simple and easy to navigate, and a log-in process involving a username and password was bypassed. The app displays large picture icons and a sizable font size that provides easy visualization. This simplicity characteristic of the app should reduce time constraints and be less burdensome to Portuguese Americans who may not be familiar with the technology.

Finally, according to the design thinking methodology, the final step of the design thinking methodology is required to complete the app development process. Diabetes apps tailored to the user culture demonstrated significant positive outcomes among their users, including reductions in HbA_1c_ levels and blood glucose concentrations, alongside increased satisfaction with the app’s functionality [15]. Previous app development highlighted the need for culturally tailored apps for diverse ethnic groups, underscoring the importance of culturally sensitive apps in diabetes management [14,15,26,27]. The development of the DiaFriend app underscores the importance of cultural considerations in health informatics. It establishes a foundation for future research in developing and evaluating culturally sensitive mobile health apps. The research team plans to address backend issues and examine the usability and acceptability of the app with Portuguese Americans with T2DM. Further details on the testing process will be provided in subsequent reports. The adaptation of such technologies has the potential to enhance self-care practices among Portuguese Americans with T2DM.

### Limitations

While the researchers successfully developed a user-centered, culturally sensitive prototype app, the current version of the DiaFriend app has several limitations due to funding and development time constraints. First, advanced functions such as real-time feedback and notifications based on users’ inputs have not been incorporated into this iteration of the app. These features could provide timely reminders to users. Timely prompts can encourage regular blood sugar monitoring, consistent carbohydrate and exercise tracking, and improved medication adherence.

Second, the app’s backend infrastructure, which requires expert development skills, has not been fully developed. This limitation hinders the app’s ability to store, process, and analyze user data securely and efficiently. Therefore, future versions of the DiaFriend app should prioritize the development of these critical backend components and integrate real-time feedback and notification features to offer a more comprehensive and user-centric diabetes self-care tool.

Third, as the research team members are not Portuguese speakers, the current version of the DiaFriend app is available only in English. This language barrier may limit the app’s accessibility and usability for Portuguese American users who prefer their native language. Previous studies highlight the need for culturally tailored apps for non-English speakers to enhance healthy eating, promote exercise, and weight control [27]. Future versions of the DiaFriend app should be translated appropriately into Portuguese to address this limitation.

Finally, the research team recognized the significance of user collaboration with designers throughout every stage of app development to ensure that the app effectively fulfills their needs. However, the COVID-19 pandemic posed challenges, limiting the ability to conduct face-to-face research with participants. Due to institutional guidelines, the research team was not permitted in-person contact with app development participants. Thus, the information about the app design derived during the ideate step was solely from the research team, which had experience caring for patients with T2DM who have limited health literacy and technological skills. Moreover, specialists such as clinical diabetes educators and endocrinologists were not included in the design and development. Thus, some valuable user and expert perspectives might have been overlooked.

The DiaFriend app version 1, which is the result of phase 2 of this app development project, will be used to revise and develop the DiaFriend app version 2. To address the limitations and enhance the functionality and usability of the DiaFriend app, the research team plans to recruit Portuguese Americans with T2DM to be involved in the remaining stages of front-end development and backend creation. Furthermore, the team plans to collaborate with a multidisciplinary health care team and experienced backend developers. This collaboration will focus on designing necessary personalized features, such as real-time feedback and notifications based on the users’ inputs, and completing the backend functionality, including data computing, analysis, and storage, before progressing to the app testing step in the design thinking methodology.

### Conclusions

As a result of phase 2 of this app development project, the prototype DiaFriend app for Portuguese Americans with T2DM was developed. In total, 2 steps in the design thinking methodology (ideate and prototype) were used to ensure the user-friendliness and cultural sensitivity characteristics of the app. The DiaFriend app version 1 comprises five features: (1) blood glucose monitoring, (2) weight tracking, (3) carbohydrate tracking, (4) exercise log, and (5) medication reminder. The carbohydrate tracking feature was explicitly tailored to correspond to Portuguese food culture. This feature includes food databases tailored to Portuguese food culture and dietary choices. The app features displayed large picture icons and a sizable font size that provides easy visualization, and the data-entry steps are easy to navigate, which should reduce time constraints and be less burdensome to Portuguese Americans who may not be familiar with the technology. Therefore, by incorporating these characteristics into the app’s design, the DiaFriend app should promote app engagement and adherence to self-management among Portuguese Americans with T2DM. The findings for this phase will serve as the basis for updating and refining the DiaFriend app, leading to the development of version 2. The usability of the app will be tested in phase 3 of this app development project, which uses the final step (test) of the design thinking methodology. The DiaFriend app lays the groundwork for subsequent studies focused on creating and assessing mobile health tools tailored to specific cultural contexts. The adoption of the DiaFriend app may significantly improve their ability to manage their condition independently. Future research could involve a randomized controlled trial comparing the use of the DiaFriend app to standard care among Portuguese Americans with T2DM, measuring outcomes such as HbA_1c_ levels and adherence to self-care behaviors. In addition, a longitudinal app development could be conducted to evaluate the long-term impact of the DiaFriend app on diabetes management and quality of life, potentially incorporating qualitative interviews to gain insights into user experiences and cultural acceptability.

## Abbreviations

HbA_1c_: glycated hemoglobin
T2DM: type 2 diabetes mellitus
